# Performance of flipped classroom teaching method during hemodialysis training of nurses

**DOI:** 10.1590/1980-220X-REEUSP-2024-0276en

**Published:** 2025-01-31

**Authors:** Zihua Lu, Jiangtao Zhu, Cheng Chen

**Affiliations:** 1 Yangzhou Polytechnic College, Department of Medical Science, 225009, Yangzhou City, Jiangsu Province, China.; 2 Shanxi Provincial Integrated TCM and WM Hospital, Department of Nephrology, 030013, Taiyuan, Shanxi Province, China.

**Keywords:** Hemodialysis, Nurse Specialists, Personal Satisfaction, Hemodiálise, Enfermeiros Especialistas, Satisfação, Diálisis Renal, Enfermeras Especialistas, Satisfacción Personal

## Abstract

**Objective:**

To compare the effectiveness of flipped classroom (FC) model with traditional lecture-based learning (LBL) model in the education of hemodialysis nurses.

**Method:**

Enrolled were 46 nurses who had received training from the hemodialysis center. These nurses were randomly assigned to FC or LBL groups, with 23 nurses in each group. FC group received the FC method, while LBL group was trained via the traditional LBL. After training, all nurses were examined for their theoretical knowledge, clinical practice skills and self-learning abilities. Furthermore, their satisfaction with the teaching mode was evaluated.

**Results:**

FC group outperformed LBL group in the mastery of theoretical knowledge. In FC group, the scores in objective, subjective and overall questions were significantly higher than those in LBL group. FC group demonstrated a superior capability in clinical practices. FC group exhibited a superior performance over the LBL group in self-learning ability. Additionally, FC group showed a higher degree of satisfaction with the training method.

**Conclusion:**

FC method benefits nurses in mastering knowledge about hemodialysis. It enhances their clinical practice skills and self-learning capabilities, and brings them with a higher degree of satisfaction.

## RESUMO

**Objetivo:**

Comparar a eficácia do modelo de sala de aula invertida (AI) com o modelo tradicional de aprendizagem baseada em palestras (ABP) na educação de enfermeiros de hemodiálise.

**Métodos:**

Foram inscritos 46 enfermeiros que receberam treinamento do centro de hemodiálise. Esses enfermeiros foram aleatoriamente designados para grupos AI ou ABP, com 23 enfermeiros em cada grupo. O grupo AI recebeu o método AI, enquanto o grupo ABP foi treinado por meio da tradicional ABP. Após o treinamento, todos os enfermeiros foram examinados quanto ao seu conhecimento teórico, habilidades de prática clínica e habilidades de autoaprendizagem. Além disso, sua satisfação com o modo de ensino foi avaliada.

**Resultados:**

O grupo AI superou o grupo ABP no domínio do conhecimento teórico. No grupo AI, as pontuações em questões objetivas, subjetivas e gerais foram significativamente maiores do que aquelas no grupo ABP. O grupo AI demonstrou uma capacidade superior nas práticas clínicas. O grupo AI exibiu um desempenho superior ao grupo ABP na capacidade de autoaprendizagem. Além disso, o grupo AI mostrou um maior grau de satisfação com o método de treinamento.

**Conclusão:**

O método AI beneficia os enfermeiros no domínio do conhecimento sobre hemodiálise. Ele aprimora as habilidades de prática clínica e capacidades de autoaprendizagem, além de proporcionar um maior grau de satisfação.

## RESUMEN

**Objetivo:**

Comparar la eficacia del modelo de aula invertida (AI) con el modelo tradicional de aprendizaje basado en ponencias (ABP) para la educación de enfermeros de hemodiálisis.

**Métodos:**

Se inscribieron 46 enfermeros que se habían capacitado en el centro de hemodiálisis. Dichos enfermeros fueron aleatoriamente designados para grupos AI o ABP, con 23 en cada grupo. El grupo AI recibió el método AI, mientras que el ABP fue entrenado mediante el tradicional ABP. Tras la formación, todos los enfermeros fueron examinadas sobre sus conocimientos teóricos, sus aptitudes para la práctica clínica y sus habilidades de autoaprendizaje. Además, se evaluó su satisfacción con el método de enseñanza.

**Resultados:**

El grupo AI superó al grupo ABP en el área de conocimientos teóricos. En el grupo AI, las puntuaciones en cuestiones objetivas, subjetivas y generales fueron significativamente superiores a las del grupo ABP. El grupo AI demostró una capacidad superior en la práctica clínica. El grupo AI superó al grupo ABP en capacidad de autoaprendizaje. Además, el grupo AI mostró un mayor grado de satisfacción con el método de formación.

**Conclusión:**

El método AI beneficia a los enfermeros en el dominio del conocimiento sobre la hemodiálisis. Mejora las habilidades de la práctica clínica y la capacidad de autoaprendizaje, además de proporcionar un mayor grado de satisfacción.

## INTRODUCTION

Worldwide, the incidence of chronic kidney disease (CKD) has been on the rise. In China, over 82 million adults with CKD had been confirmed in 2019^(1)^. In a considerable number of patients, CKD may progress into end-stage kidney disease (ESKD), requiring kidney replacement therapy. Globally, about 759 per million patients need kidney replacement therapies, including hemodialysis, peritoneal dialysis, and kidney transplantation. In China, hemodialysis is the predominant therapy for ESKD, serving over 500,000 patients^(2)^.

Hemodialysis nurses undertake a pivotal role in performing hemodialysis for ESKD patients^(3)^. However, there is a significant shortage of qualified professionals in this field. A proficient hemodialysis nurse can only be established on a good mastery of professional knowledge, skills and related subjects, thus highlighting the necessity of a better teaching methodology for training hemodialysis nurses. In China, most tertiary A hospitals just focus on clinical practices, rather than the teaching of nursing. Lacking highly efficient training programs, hemodialysis nurses or nursing students may face obstacles in the development of critical thinking, self-learning and clinical skills^(2,4)^. Consequently, the use of advanced educational strategies to train hemodialysis nurses are imperative.

Flipped classroom (FC), coined by Bergmann and Sams in 2007, provides active learning strategy^(5)^. In recent years, FC has been widely used and demonstrated effective outcomes in nursing education^(6, 7, 8)^. Compared to traditional lecture-based learning (LBL), FC has been shown to enhance active learning, which places the student at the heart of the learning process, to train nursing students’ critical thinking, decision making, and clinical reasoning^(9)^. The LBL is teacher-centered, and the student is not active in their learning process. The FC reverses the LBL mode, emphasizing students’ preparation before class and shifting the power of initiative and decision-making from the teacher to the students^(10)^.

Up to now, the effectiveness of FC compared with LBL has been established^(11)^. However, no studies have evaluated the outcomes of FC in training hemodialysis nursers. Our study compared FC with LBL methods in terms of theoretical knowledge, practical skills and self-learning ability, and other relevant competencies for hemodialysis nursing.

## METHOD

### Study Participants Materials and Method

This study selected 46 female nurses from various hospitals in Shanxi Province, China, who had no prior experience in hemodialysis. They received hemodialysis training at the Hemodialysis Center at Shanxi Province Integrated TCM and WM Hospital from August to December 2022.

The nurses were randomly allocated into FC group and LBL group, with 23 in each group. Both groups were given the same number of training sessions, totaling 60 sessions, and completed the training within 5 months (from August to December 2022). Head nurse of the hemodialysis center, a nurse with extensive experience in FC teaching, served as the primary educator. None nurses were familiar with the primary educator.

Inclusion criteria included: (1) Holding a bachelor’s degree in nursing; (2) Receiving instruction from the same educator; (3) Employing uniform assessment standards. Exclusion criteria included: (1) Refusing to participate in the study for various reasons; (2) Absence of a nursing qualification certificate.

### LBL Group

Nurses assigned to LBL group underwent traditional LBL training, led by the head nurse of the hemodialysis center who was the primary instructor. The curriculum covered foundational aspects of hemodialysis, including hemodialysis principles, arteriovenous fistula puncture, arteriovenous fistula care, semi-permanent or clinical catheter care, and diagnosis and management of common dialysis-related discomfort or symptoms. Instruction was delivered primarily through slide presentations. Additionally, basic clinical practical skills were taught during real-time settings by instructors.

### FC Teaching Group Observation Indicators

In the FC group, the head nurse of the hemodialysis center was also the primary educator. The lecture materials were disseminated to the nurses one week prior to each lesson by Wechat Software.

The educator explained key concepts and difficulties presented by students, followed by nurse-led discussions. Unresolved issues were explained by the teacher, who acted as mentor and facilitator, to enhance interaction with the students.

On the other hand, instructional videos detailing basic operational skills were provided to the students one week prior to their actual lesson for pre-class review. The practical sessions began with discussion on these practical skills. Following that, the instructor conducted bedside demonstrations of specific skills discussed before.

### Observation Indicators

**Theoretical Knowledge:** Subsequent to the entire training, nurses’ mastery of theoretical knowledge for hemodialysis was assessed through a written examination, with a maximum score of 100, including subjective (40 points) and objective (60 points) components.

The exam consisted of 60 single-choice questions and 4 essay questions. Each single-choice question was given a score of 1 point, and each essay question a score of 10 points. The content covered fundamental theories related to hemodialysis. The exam was designed by the head nurse of the hemodialysis center. A higher score reflected a better mastery.

**Clinical Skills:** After the training was completed, assessment was conducted to evaluate the clinical practical skills in accordance with the requirements of the Standard Operating Procedures for Blood Purification (2021 Edition) issued by the National Health Commission of China, including preparation prior to hemodialysis, aseptic technique, arteriovenous fistula puncture and its management, temporary or semi-permanent catheters management, and maintenance of the hemodialysis machine post-treatment. Each item was assessed on a score from 1 to 10, with a higher score representing a better performance in practical operation.

**Self-Directed Learning:** Subsequent to the theoretical knowledge test, nurses’ self- directed learning capability was assessed using the scale designed by Shanxi University of Traditional Chinese Medicine in 30 minutes, focusing on self-management ability, learning motivation, formulation and execution of learning plans, and active learning ability. The scale was filled out anonymously. Each item was evaluated as scores ranging from 1 to 10, where a higher score represented a more pronounced competence.

**Satisfaction with Training:** After the self-directed learning scale was completed, participants were required to complete questionnaires anonymously about teaching effectiveness and personal experience. Responses were gathered from 1 to 5, in which 1 indicated very dissatisfied, and 5 extremely satisfied.

## Ethical Aspect

The current research was authorized by the Medical Ethics Committee of the Shanxi Province Integrated TCM and WM Hospital (approval no. KYLL-092). All participants have signed the Informed Consent Form.

## Statistical Analysis

Data analysis was performed using the SPSS software. Data adhering to a normal distribution were expressed as mean ± standard deviation. Differences between two groups were analyzed by the independent sample T-test. Comparisons between proportions were performed with the chi-square test. P < 0.05 was the threshold for statistical significance.

## RESULTS

### Participants’ Demographic Data

The demographic data of FC group and LBL group are shown in (Table 1). This study included 46 nurses within two groups: FC group (n = 23) and LBL group (n = 23). All participants in this study were female. The average age of nurses in the FC group was 25.10 ± 0.90 years, and 24.64 ± 0.70 years in the LBL group. In the FC group, there were 11 nurses from tertiary hospitals and 12 from secondary hospitals. In the LBL group, there were 10 nurses from tertiary hospitals and 13 from secondary hospitals, with no statistically significant difference. All 46 nurses had experience in medical nursing, with the FC group having an average work duration of 28.17 ± 4.37 months and the LBL group 28.95 ± 3.57 months, showing no statistically significant difference.

**Table 1:** Demographic data of participants – Taiyuan, Shanxi Province, China, 2022.

Characteristics	FC group	LBL group	t/χ^2^	P
Age (year)	25.10 ± 0.90	24.64 ± 0.70	1.912	0.062
Hospital grade			0.088	0.760
Tertiary hospital [n (%)]	11 (47.82%)	10 (43.47%)		
Secondary hospital [n (%)]	12 (52.18%)	13 (56.53%)		
Work experience (months)	28.17 ± 4.37	28.95 ± 3.57	-0.664	0.510

### Efficiency of FC in Improving Theoretical Knowledge

**Figure 1:**
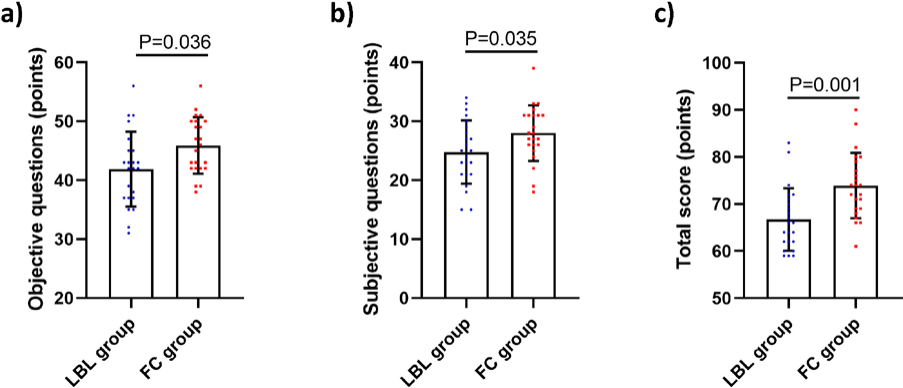
Comparison of the results of theoretical knowledge assessment between two groups of students. (A) Scores of objective questions in the assessment; (B) Scores of subjective questions in the assessment scores; (C) The total score of theoretical knowledge assessment.

FC group exhibited significantly higher scores in objective and subjective questions, as well as the overall theoretical knowledge, compared to LBL group (Figure 1).

### Efficiency of FC in Improving Clinical Skills

**Figure 2:**
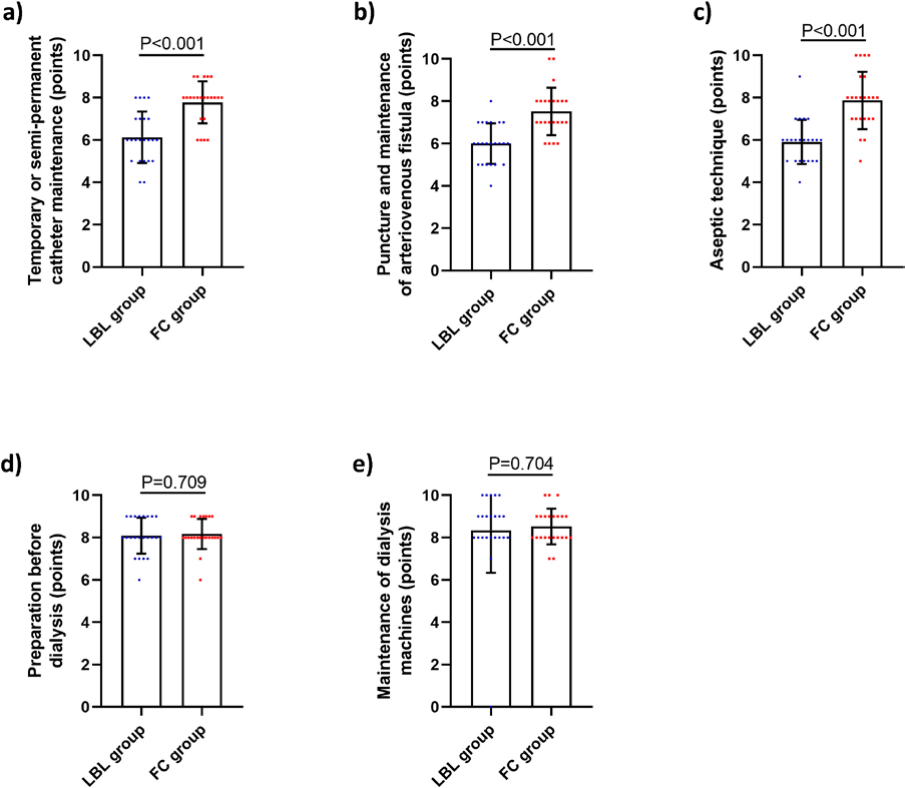
Comparison of the results of clinical skills assessment between two groups of students. (A) Scores of management of temporary or semi-permanent catheters assessment; (B) Scores of arteriovenous fistula puncture and management assessment; (C) Scores of aseptic technique assessment; (D) Scores of pre-hemodialysis preparation assessment; (E) Scores of maintenance of the hemodialysis machine assessment.

In FC group, the scores in critical items like aseptic technique, arteriovenous fistula puncture and management, as well as the management of temporary or semi-permanent catheters, were significantly superior to those in LBL group, with significant differences. Conversely, FC group showed higher scores in pre-hemodialysis preparation and post-hemodialysis maintenance of the hemodialysis machine, but with no significant differences (Figure 2).

### Efficiency of FC in Improving Self-Learning Ability

**Figure 3:**
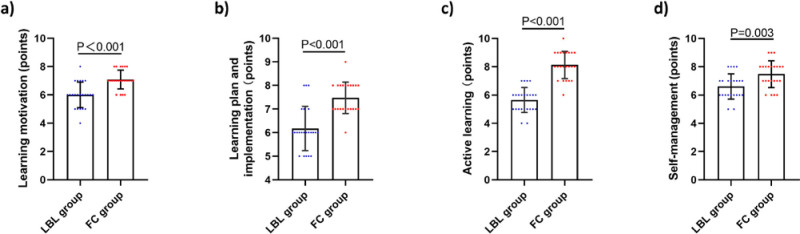
Comparison of the results of self-learning ability assessment between two groups of students. (A) Scores of learning motivation assessment; (B) Scores of learning plan and implementation assessment; (C) Scores of active learning assessment; (D) Scores of self-management assessment.

Compared to the LBL group, the FC group demonstrated significantly superior performances across the following items: self-management, learning motivation, formulation and execution of learning plans and active learning (Figure 3).

### Satisfaction with FC

**Figure 4:**
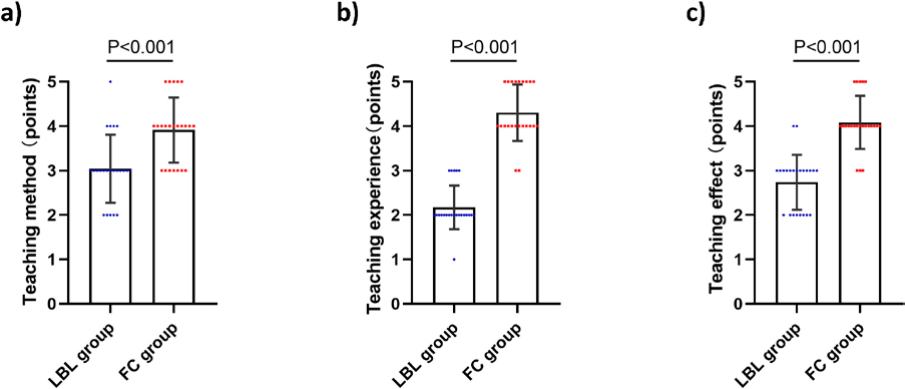
Comparison of the results of Satisfaction with FC assessment between two groups of students. (A) Scores of teaching method assessment; (B) Scores of teaching experience assessment; (C) Scores of teaching effect assessment.

Regarding teaching satisfaction, FC group showed a substantially higher satisfaction with FC teaching, its effectiveness and overall educational experience compared to the LBL group (Figure 4).

## DISCUSSION

Hemodialysis, a critical component in nephrology, has its own characteristic practices and protocols. Nursing staff plays a critical role in daily operation of hemodialysis. The expertise of hemodialysis nurses is a determinant of the outcomes of dialysis. Hemodialysis-specific knowledge includes the foundational knowledge of hemodialysis, aseptic techniques, arteriovenous fistula puncture and its management, and management of clinical or semi-permanent catheters. Given that hemodialysis often represents a lifelong treatment for a majority of patients, these individuals often experience physiological discomfort and psychological distress, probably turning them resistant towards the care and treatment undertaken by the nursing staff^(12)^. Compared to the standard ward, nursing training is impossible to be performed in a hemodialysis room, which presents a complex environment. In conventional LBL, teachers are very active in the teaching process, while students often play a more passive role, leading to deficiency in students’ self-directed learning of theoretical knowledge and practical skills^(13)^. This teaching mode is particularly not suitable for the education of hemodialysis nursing. In the present study, FC was proven as an innovative and effective instrument to improve the training outcomes of hemodialysis nurses.

FC teaching is student-centered, well adapted to trends of modern education^(9,14,15)^. This method encourages students to learn independently, so that they may actively raise questions and play more prominent roles in learning process^(16,17)^. As a pedagogical model prevailing in this information age, FC has revolutionized teaching process, auxiliary teaching methods, and instructors’ roles. FC has been extensively adopted in medical and nursing education in recent years. Yang et al.^(18)^ have demonstrated that compared to LBL, FC notably improves medical students’ comprehension and critical thinking skills. Farina et al.^(6)^ have found that FC can notably improve nursing graduate students’ satisfaction with anesthesiology courses, and their academic achievements. Similarly, Liu et al.^(19)^ have reported that FC significantly enhances the self-learning capability of newly recruited nurses. Except for medical students or nursing students, FC teaching can increase the effectiveness of teaching, as well as the clinical diagnosis and treatment ability in the standard training for resident physicians^(20)^.

Our study revealed that the scores in objective, subjective, as well as overall theoretical knowledge in the FC group were significantly higher than those in the LBL group, suggesting that FC teaching method can help nurses better grasp the theoretical knowledge of hemodialysis, which is consistent with the findings of Li et al.^(21)^. Furthermore, FC group showed superior proficiency in aseptic technique, arteriovenous fistula puncture and management, and management of temporary and semi-permanent catheters. It is clear that the FC teaching method can significantly enhance clinical practical skills of nurses. Zhang et al.^(22)^ have reported that the FC teaching method improves clinical skills in medical students. Wilson and Hobbs^(23)^ have found that the FC teaching method facilitates prelicensure nursing students in mastering fundamental nursing skills.

Our findings are consistent with previous literature, providing further strong evidence for the conclusion that the FC teaching method enhances clinical practical skills of nurses. He et al.^(24)^ have found that, compared to the LBL teaching method, the FC teaching method can improve self-directed learning abilities in pharmacy students. Rui et al.^(25)^ have confirmed that the FC teaching method can enhance medical students’ interest in learning electrocardiograms and their self-directed learning abilities. Joseph et al.^(26)^ have found that compared with the didactic lecture format, FC strategy improves nursing students’ satisfaction with an anatomy or physiology course. In present study, compared to LBL group, the FC group exhibited a stronger self-learning ability and a higher level of satisfaction with the teaching mode and outcomes.

## LIMITATIONS

This study has a number of limitations. Firstly, this was a single-centre study with a limited sample size. Secondly, the design did not consider the participants’ personal characteristics (i.e. personality and academic performance in college). Therefore, in addition to these confounding factors, other variables such as long-term career planning, interest in the field of hemodialysis, and work experience, may also influence the results.

## CONCLUSION

FC method is highly effective for hemodialysis nurses to improve the mastery of theoretical knowledge and practical skills, enhance their self-learning ability, and improve their overall satisfaction with learning process and outcomes. Further investigations are warranted to validate its performances in nursing education.
